# First designing of a silicene-based optical MOSFET with outstanding performance

**DOI:** 10.1038/s41598-023-33620-2

**Published:** 2023-04-21

**Authors:** Hamed Emami-Nejad, Ali mir, Zeinab Lorestaniweiss, Ali Farmani, Reza Talebzadeh

**Affiliations:** 1 Faculty of Engineering, Lorestan University, Khorramabad, Iran; 2 Faculty of Science, Lorestan University, Khorramabad, Iran

**Keywords:** Materials science, Nanoscience and technology, Optics and photonics

## Abstract

Miniaturized integrated optical devices with low power consumption have long been considered hot candidates for plasmonic applications. While 2D materials such as graphene have been proposed for this purpose, they suffer from large propagation loss and low controllability at room temperature. Here, a silicene-based optical MOSFET with excellent performance is designed to achieve integrated circuit optical technology. The designed device is comprised of a silicene optical waveguide whose switching operation is performed by a gate and has a structure similar to an enhancement MOSFET with a formed channel. Unlike graphene, the surface conductivity of silicene can be controlled by both chemical potential and an electric field perpendicular to its surface. This unique feature of silicene is used to design and simulate an optical-MOSFET with transverse electric polarization at 300 K. The salient characteristics of the optical device include its nanoscale dimensions, ultra-low insertion loss of 0.13 dB, infinite extinction ratio, and quality factor of 688, proposing it as a promising tool for optical integration.

## Introduction

Optical telecommunications employing semiconductor lasers as the light sources, and optical fibers as the transmission media are the only current solution for managing and massive growth of telecommunication and data traffic. A fiber optic can provide a bandwidth of 25 THz, and a cable comprising ~ 1,000 optical fibers is capable of carrying 6 billion video calls simultaneously. New services such as high-definition television and network computing have been introduced, increasing the demand for high bandwidth networks. The potential bandwidth of these optical fibers can be fully realized by developing other components of the optical network system, (e.g., detectors, multiplexers, buffers, and switches), thereby allowing for a match of the transmission rate and bandwidth. In general, optical switches are related to the routing of message information in response to regulatory control signals. The message information can be in large blocks of multiple traffic in the optical core network or be in a large number of lower-bit channels for delivery to users in the optical network. However, the use of an optical switch may not only be limited to communication networks but may also include the communication cores of a large multiprocessor computer with a data rate exceeding 100 Gbps. By experimenting with new designs that use quantum phenomena for secure communication and computing, new architectures would be needed for switches without interrupting the basic information of quantum packets^1^.

Based on their structure and operation, optical switches have different types, the most important of which are electro-optical^2^, thermal-optical^3^, magnetic-optical^4^, MEMS-based^5^, SOA-based^6^, and photonic crystals –based^7^ switches. In addition to the aforementioned technologies, it is possible to realize a new type of optical switch in optoelectronics^8^ when it comes to the advent of 2D materials such as graphene with the capability of controlling the density of highly mobile electrons on the surface.

The discovery of graphene has greatly motivated the research on graphene-like 2D materials, including hexagonal boron nitride (h-BN)^9^, silicene^10^, germanene (i.e., hexagonal networks of silicon and germanium)^11^, stanene^12^, phosphorene^13^, and chemical constituents of transition metals with the typical formula of MX_2_^14^. Among these materials, silicene has received more attention due to its compatibility with current technology, as well as its interesting electrical and optical properties. However, silicene does not have a layered bulky counterpart such as graphite, so the synthesis of this 2D material requires a bottom-up method (e.g., epitaxial growth on a substrate)^15^.

The theoretical study of silicene goes back to an article by Takeda and Shiraishi on the hexagonal rings of silicon and germanium, referred to as their carbon counterpart^16^. One of the most important 2D structures is silicene, in which the presence of massless Dirac fermions has been predicted. Moreover, silicene has shown a spin-quantum effect^17^. Nevertheless, the major challenge facing silicene is that it cannot remain stable in air, and reacts easily with oxygen atoms, thereby producing Si–O^18–20^. So far, some approaches such as silicene hydrogenation and encapsulation have been proposed to solve this problem^21^. In the silicene hydrogenation approach, nitrogen atoms are bonded to silicon atoms, thus preventing silicene from reacting with oxygen.

On the other hand, silicene can be encapsulated between Al_2_O_3_^19,20^ and^22^, h-BN^23^ and^24^, and graphene^25^. The authors in^20^ have developed a method that preserves silicene during the fabrication and transfer processes of the device. In this case, using Al_2_O_3_ during the fabrication process, silicene was encapsulated to prevent its reaction with air. This method can also be applied to other materials (germanium, phosphorus, etc.).

Generally, in addition to Al_2_O_3_, h-BN is also used between silicene and oxide layers to maintain the structure, high carrier mobility^26^, and optical properties of silicene^27^. The h-BN layer as a buffer is important in the fabrication of devices based on intrinsic silicene^24^. The presence of an h-BN layer on silicene prevents the formation of covalent bonds. In addition, both silicene and h-BN have been observed to retain their structure and remain intact when they are in contact with each other. This stands true when these layers are exposed to a vertical electric field. By inserting an h-BN layer into the silicene-based device, one can maintain structural symmetry. Since the interactions between silicene and h-BN are of the van der Waals type, it is possible to use materials such as silicon carbide (SiC) and graphene to act as h-BN in silicene-based devices^28^–^30^.

Apart from the studies performed on silicene encapsulation, other investigations into the optical and electrical properties of silicene nanoribbons and silicene nanotubes have been reported in the literature^31,32^. Research on the types of silicene buckling (i.e., low and high buckling) and its properties has also been performed^33^. Due to the challenges mentioned above, silicene has been proposed as a preferred material in recent decades, providing integration with silicon-based technology. This integration is one of primary advantages of silicene as a material for future electronic devices with the availability of a bulk infrastructure for the silicon technology. Silicene has been utilized to design several electronic and optical devices, ranging from metal–oxide–semiconductor field-effect transistors (MOSFETs) to optical detectors for use in molecular sensors.

Various configurations of silicene-based FETs have been reported, the most important of which are dual-gated silicene FET, silicene-based FET with alkaline absorbers, silicene nanoribbon-based FET, Z-shaped silicene nanoribbon FET, silicene nanotube-based FET, and Li-Cl co-decorated silicene FET.

Ni et al. investigated a silicene FET with a dual-gate configuration in which the silicene layer sandwiched between h-BN buffer and SiO_2_ dielectric layers^24^, allowing for the control of both the doping level and the electric field perpendicular to the silicene surface. The ON/OFF ratio of the resulting device current was obtained to be about 4.3 at room temperature, which is a very low value due to the direct effect of the short channel of the device. In Ref.^26^, the authors designed a FET consisting of a central region and two electrodes made of silicene using the adsorption of Na atoms on the silicene surface. The dielectric layer was made of SiO_2_, and an h-BN layer was used in the structure to prevent the interaction between silicene and SiO_2_. In this case, the ON/OFF ratio reached about 4 × 10^8^. Alternatively, Selimian et al. designed a FET using silicene nanorods^34^, outperforming FETs fabricated based on graphene nanorods. Li et al. also reported a FET based on Z-shaped silicene nanoribbons, having an ON/OFF ratio of about 10^6^^35^.

In another investigation, silicene nanotubes were employed to fabricate a FET with an ON/OFF ratio of about 10^7^, which is higher than that of graphene counterpart^36^. In Ref.^23^, researchers have proposed a new method for designing a high-performance FET channel by decorating silicene with Li and Cl. The electrodes and channel of the device were made of silicene and Li-Cl co-decorated silicene, respectively, giving rise to an ON/OFF ratio of 4.66 × 10^4^.

By using an ion-sensitive field-effect transistor (ISFET) structure with silicene layers and electrolyte, Sazzadur Rahman et al. have designed a sensor with the capability of label-free detection of deoxyribonucleic acid (DNA) for disease-related gene expression^37^. Also, Kharadi et al. reported an optical detector using silicene-MoS_2_ heterojunction with high efficiency^38^. This was the first time that the silicene-MoS_2_ heterojunction had been investigated as a high-performance device for optical detector applications. Elsewhere, two optical switches have recently been proposed based on silicene. The first one was a plasmonic electro-optical switch based on a silicene waveguide encapsulated between layers of Al_2_O_3_^39^, providing a better performance compared to its graphene counterpart. The second switch was designed based on metamaterials, utilizing graphene-silicene-graphene (G-S-G) structure to control the switching of the transmitted light^40^.

Silicene has unique properties compared to graphene^41^, the most important advantage of which is the tunability of the band gap with an external field perpendicular to the surface. However, no attention has been paid to the design of a silicene-based optical MOSFET (OMOSFET), according to the best of our knowledge. In this essay, a plasmonic switch is designed and employed as an OMOSFET using silicene that emits surface plasmon polaritons (SPPs) in transverse electric (TE) mode. Electromagnetic waves propagating along the surface between a metal (or semiconductor) and a dielectric are generally in the infrared range^42^. Metals such as gold and silver are commonly used to emit surface plasmon polaritons (SPPs)^43^. In recent years, graphene has been used to emit SPPs^44^, outperforming gold and silver in terms of adjustability and confinement limit with fewer losses.

While conventional MOSFETs use a gate to control and transmit the electrical charge, our proposed OMOSFET uses a gate to control (turn on or off) an optical signal with TE polarization. Therefore, the proposed device can be a suitable candidate for designing optical processors that use optical signals instead of electrical signals for data processing, thereby avoiding overheating problems and having the capability to work at THz speeds. The structure of the article is outlined as follows: In section "Physical structure of the proposed device", we describe the physical structure and performance of the proposed device. In sections "Device modeling"‒“Switching performance”, we discuss the mathematical relations of the problem and present the results of the numerical simulation. In section "Fabrication process", we analyze the evaluation of the switching performance of the proposed device, and finally, in the last section we have presented the method of fabrication the proposed device.

## Physical structure of the proposed device

**Figure 1:**
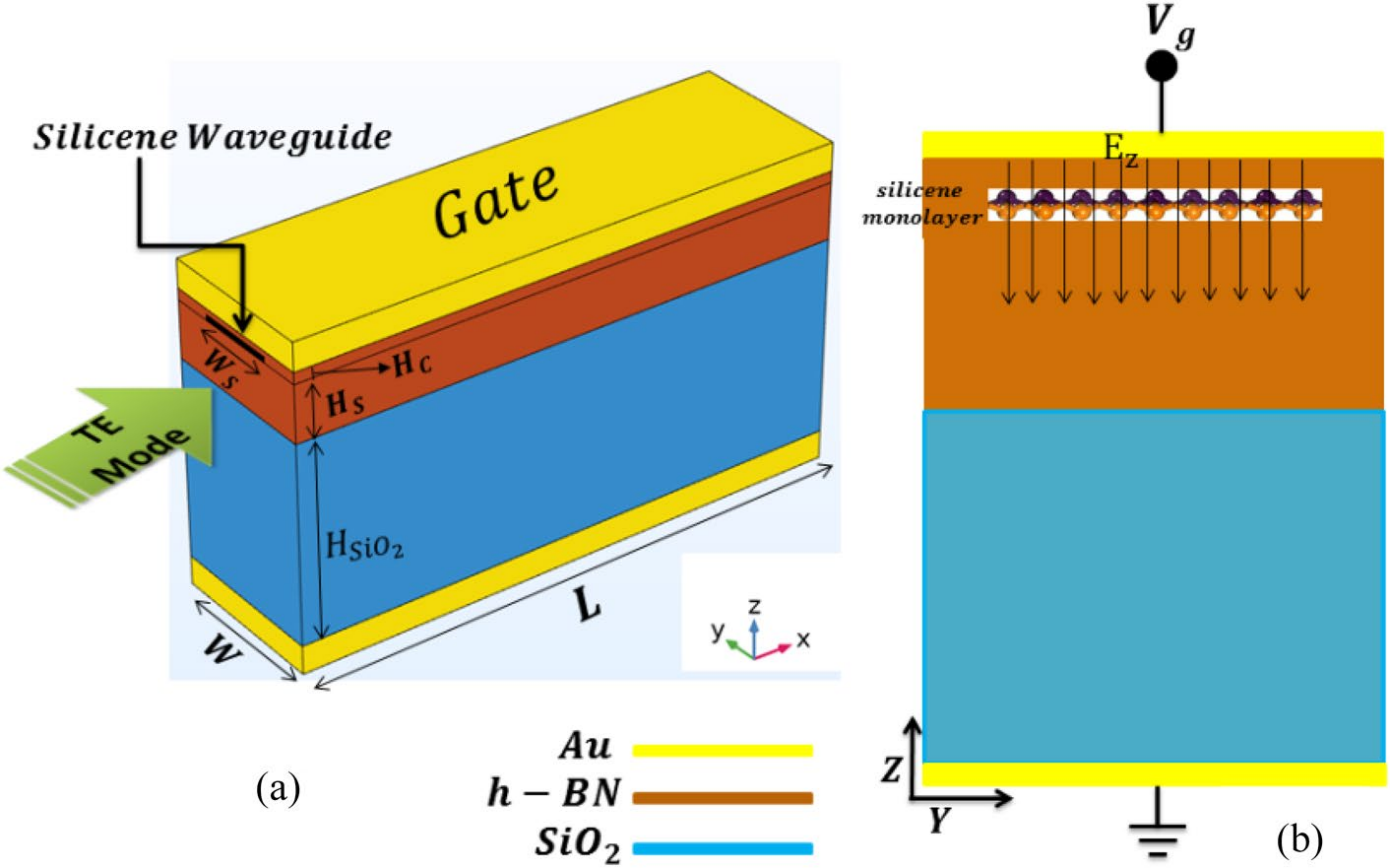
Structure of the proposed device: (**a**) 3D model with W_s_ = 150 nm, W = 300 nm, L = 500 nm, H_SiO2_ = 300 nm, H_c_ = 5 nm, and H_s_ = 100 nm; and (**b**) cross-sectional view with an applied electrostatic field through the gate terminal.

Figure 1 shows the design of the proposed OMOSFET. A 3D view of the optical switch is depicted in Fig. 1(a). In this switch, a silicene waveguide is encapsulated between two h-BN layers. The bottom h-BN layer has a thickness of 100 nm, acting as both the silicene substrate and buffer to prevent the silicon atoms from reacting with SiO_2_. The 5 nm-thick top BN layer is employed as both the protective layer for the silicene monolayer (thus preventing silicon atoms from reacting with air) and the dielectric gate. Figure 1(b) shows a cross-section of the proposed switch. In this figure, the operation of the optical switch is presented. The structure of this switch is similar to an enhancement-MOSFET. By applying a suitable voltage to the gate, a constant electric field perpendicular to the silicene surface is created, providing the necessary conditions for the transmission of TE waves. When the gate voltage is cut off, the TE wave can no longer be transmitted due to the zero electric field.

## Device modeling

The proposed OMOSFET operates in the TE mode. The electric vector (E) in the TE mode is always perpendicular to the propagation direction. In general, the TE mode types are defined as TE_m,n_, where m and n are the numbers of half-wave patterns across the width and height of the waveguide, respectively. The numbers m and n can always have integer values from zero to infinity, indicating the wave modes inside a waveguide^45^. The electric field and magnetic field components of TE waves are defined as follows^46^:

1 $$ \vec{H} = (\vec{H}_{X} ,\,0,\,\vec{H}_{Z} ),\,\vec{E} = (0,\,\vec{E}_{Y} ,\,0) $$

and

2 $$ \vec{{\rm E}}_{{\rm y}}  = \left\{ {\begin{array}{*{20}c}    {{\rm A}_{1} {\rm e}^{{ - \upgamma _{1} {\rm z}}} ~~~~~0 < z < \infty }  \\    {{\rm A}_{2} {\rm e}^{{\upgamma _{2} {\rm z}}} ~~~~ - \infty  < z < 0}  \\   \end{array} } \right.,\vec{{\rm H}}_{{\rm x}}  = \frac{i}{{\upmu _{ \circ } \upomega }}\left\{ {\begin{array}{*{20}c}    { - {\rm B}_{1} \tfrac{{\upgamma _{1} }}{{\upmu _{1} }}{\rm e}^{{ - \upgamma _{1} {\rm z}}} ~~~~~0 < z < \infty }  \\    {{\rm B}_{2} \tfrac{{\upgamma _{2} }}{{\upmu _{2} }}{\rm e}^{{\upgamma _{2} {\rm z}}} ~~~~ - \infty  < z < 0}  \\   \end{array} } \right.,\vec{{\rm H}}_{z}  = \frac{i}{{\upmu _{ \circ } \upmu _{i} \upomega }}\frac{{{\rm d}\vec{{\rm E}}_{y} }}{{{\rm dx}}} $$

where γ_i_ is the decay constant and given by: $\gamma_{i} = q_{ \circ } \sqrt {q^{2} - \varepsilon_{i} \mu_{i} }$, in which q is the propagation constant and q_0_ is the wave vector in the free space ($q_{ \circ } = \omega /c$). Since the silicene waveguide in the proposed device is surrounded by the h-BN layer, one can obtain Eq. (3) by applying boundary conditions ($\vec{E}_{y1} = \vec{E}_{y2} ,{ }\vec{H}_{x1} - \vec{H}_{x2} = \sigma_{s} \vec{E}_{y} ,{ }\vec{B}_{1} = \vec{B}_{2}$) and solving Maxwell's equations as follows:

3 $$ \frac{i}{{\omega \mu_{ \circ } }}\left( {\vec{B}_{1} \frac{{\gamma_{1} }}{{\mu_{1} }} + \vec{B}_{2} \frac{{\gamma_{2} }}{{\mu_{2} }}} \right) = \sigma_{s} \vec{E}_{y} $$

The dispersion relation for the TE wave is expressed below:

4 $$ \frac{{\gamma_{1} }}{{\mu_{1} }} + \frac{{\gamma_{2} }}{{\mu_{2} }} = i\sigma_{s} \omega \mu_{ \circ } $$

Since $\mu_{hBN}^{1} = \mu_{hBN}^{2} = 1$ and $\gamma_{hBN}^{1} = \gamma_{hBN}^{2} = \gamma$, Eq. (4) is simplified as follows:

5 $$ 2\gamma - i\sigma_{s} \omega \mu_{ \circ } = 0 $$

where $\sigma_{s}$ is the surface conductivity of silicene and ω is the angular frequency. The above equation is satisfied when the imaginary part of $\sigma_{s}$ becomes negative, thereby initially requiring the calculation of $\sigma_{s} = \sigma_{{{\text{intra}}}} + \sigma_{{{\text{inter}}}}$. The intra-band and inter-band conductivity contributions are obtained from the following relations^47^:

6 $$ \sigma_{{{\text{int}} ra}} (\omega ,\,\,\varsigma ,\,\,\eta ,\,\,E_{ \bot } ,\,\mu_{c} )\, = \mathop \int \nolimits \frac{{ie^{2} e^{{\tfrac{1}{\int }}} ((\mu_{c} + \tfrac{kT}{\int })^{2} - \tfrac{1}{4}\Delta [\varsigma ,\eta ]^{2} )}}{{4\int^{2} \pi \hbar^{2} (1 + e^{{\tfrac{1}{\int }}} )^{2} (\mu_{c} + \tfrac{kT}{\int })(\omega + \tfrac{i\Gamma }{\hbar })}} + \frac{{ie^{2} e^{{ - \tfrac{1}{\int } - \tfrac{{2\mu_{c} }}{kT}}} ((\mu_{c} + \tfrac{kT}{\int })^{2} - \tfrac{1}{4}\Delta [\varsigma ,\eta ]^{2} )}}{{4\int^{2} \pi \hbar^{2} (1 + e^{{ - \tfrac{1}{\int } - \tfrac{{2\mu_{c} }}{kT}}} )^{2} (\mu_{c} + \tfrac{kT}{\int })(\omega + \tfrac{i\Gamma }{\hbar })}} d\int $$

7 $$ \begin{gathered} \sigma_{{{\text{int}} er}} (\omega ,\,\varsigma ,\,\eta ,\,E_{ \bot } ,\,\mu_{c} ) = \mathop \int \nolimits \frac{{ie^{2} \omega (\tfrac{{{\text{Sinh}} [\tfrac{1}{\int }]}}{{{\text{Cosh}} [\tfrac{1}{\int }] + {\text{Cosh}} [\tfrac{{\mu_{C} }}{kT}]}}}}{{16\pi kT( - \tfrac{{4k^{2} T^{2} }}{{\int^{2} }} + \omega^{2} \hbar^{2} )}} - \frac{{\tfrac{{{\text{Sinh}} [\tfrac{\omega \hbar }{{2kT}}]}}{{{\text{Cosh}} [\tfrac{{\mu_{C} }}{kT}] + {\text{Cosh}} [\tfrac{f\pi \hbar }{{kT}}]}}(\tfrac{{4k^{2} T^{2} }}{{\int^{2} }} + \Delta [\varsigma ,\eta ]^{2} )}}{{16\pi kT( - \tfrac{{4k^{2} T^{2} }}{{\int^{2} }} + \omega^{2} \hbar^{2} )}}d\int \ominus \hfill \\ + \frac{{e^{2} \theta [\omega ,\varsigma ,\eta ]{\text{Sinh}} [\tfrac{\omega \hbar }{{2kT}}](1 + \tfrac{{\Delta [\varsigma ,\eta ]^{2} }}{{\omega^{2} \hbar^{2} }})}}{{16\hbar ({\text{Cosh}} [\tfrac{{\mu_{C} }}{kT}] + {\text{Cosh}} [\tfrac{\omega \hbar }{{2kT}}])}} + \frac{{ie^{2} }}{4\hbar }\frac{{{\text{Sinh}} [\tfrac{\omega \hbar }{{2kT}}]}}{{{\text{Cosh}} [\tfrac{{\mu_{C} }}{kT}] + {\text{Cosh}} [\tfrac{\omega \hbar }{{2kT}}]}}(\frac{\Delta [\varsigma ,\eta ]}{{2\omega \pi \hbar }} - \frac{{Log[Abs[\tfrac{\omega \hbar + \Delta [\varsigma ,\eta ]}{{\omega \hbar - \Delta [\varsigma ,\eta ]}}]](1 + \tfrac{{\Delta [\varsigma ,\eta ]^{2} }}{{\omega^{2} \hbar^{2} }})}}{4\pi }) \hfill \\ \end{gathered} $$

8 $$ \Delta [\varsigma ,\eta ,\,E_{ \bot } ] = |eE_{ \bot } d - \varsigma \eta \lambda_{SO} | $$

where $\varsigma = + 1( - 1)$ and $\eta = + 1( - 1)$ are the electron spin up (spin down) index, and k(k') valley electrons index. $E_{ \bot }$ is the electric field perpendicular to the silicene surface, and ${\upmu }_{C}$ is the chemical potential (Fermi energy) of silicene. Furthermore, e, k, T, $\epsilon$ and ћ denote the electron charge, Boltzmann's constant, temperature, electrical energy, and Planck's constant, respectively. $\Gamma = 10^{ - 5} eV$ is the phenomenological dispersion rate and $\theta [\omega ,\xi ,\eta ]$ is the Heaviside function. $\Delta [\varsigma ,\eta ,\,E_{ \bot } ]$ represents the band gap amplitude of silicene, being dependent on $\varsigma$, $\eta$, and $E_{ \bot }$. d = 0.46 Å is the vertical distance between silicene sublattices, and $\lambda_{SO} = 3.9\,\,meV$ represents the spin–orbit coupling strength of silicene.

**Figure 2:**
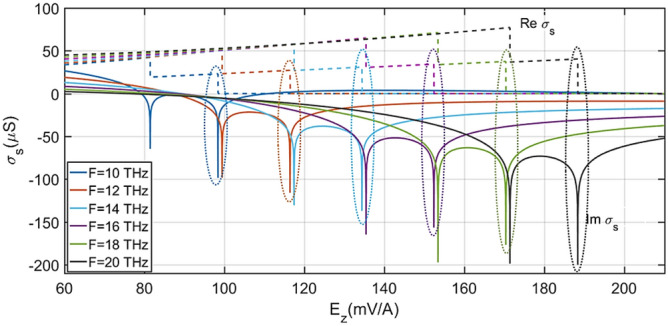
Variations of real and imaginary parts of silicene surface conductivity as a function of Ez for different frequencies. The graphs with dotted and solid lines show the real and imaginary parts, respectively.

As can be inferred from the above relationships, the surface conductivity of silicene, unlike graphene, can be controlled by $E_{ \bot }$, in addition to being controllable the chemical potential. In Ref.^48^, the comparison between emission regimes of TE waves of silicene and graphene has been investigated. Ukhtary et al. showed that by applying $E_{ \bot }$, TE waves can be propagated in a larger frequency range for silicene. While the TE wave propagation range for graphene is only possible in a narrow frequency range of $1.667E_{F} < \hbar \omega < 2E_{F}$^49^, it can be increased for silicene by increasing $E_{ \bot }$. Hence, an OMOSFET was designed in the present study using this feature of silicene. According to.the structure shown in Fig. 1, $E_{ \bot } = E_{z}$ is created by the gate terminal to control $\sigma_{s}$ and fulfill Eq. (5). As a result, $E_{z}$ is considered to be the main variable. Figure 2 shows the changes in the real and imaginary parts of $\sigma_{s}$ for $E_{z} = 60 - 210mV/$Å. These values were investigated for different frequencies in the range from 10 to 20 THz at the temperature of 300 K.

According to $1/\gamma = 2/i\omega \sigma_{s} \mu_{0}$ for TE waves^49^, where $1/\gamma$ is the optical confinement length and $\mu_{0}$ is the vacuum permeability, better propagation of surface waves occurs at smaller values of $1/\gamma$. As a result, larger values of the imaginary part of conductivity (${\text{Im}} \,\sigma_{s}$) provide smaller values of the confinement length. In addition, TE surface waves are not damped when ${\text{Re}} \,\sigma_{s} = 0$^46^. Based on these key points, the best performance in the transmission of TE waves occurs when ${\text{Re}} \,\sigma_{s} = 0$, and ${\text{Im}} \,\sigma_{s}$ is a large negative number (> − 60 µS). Thus, the ranges marked by dotted lines in Fig. 2 are taken into account.

h-BN is a uniaxial hyperbolic material with a tensor dielectric function. The performance of h-BN is characterized by the two following dielectric functions: one along the symmetry axis of the crystal ($\varepsilon_{z} (\omega )$), and the other in the base plane ($\varepsilon_{ \bot } (\omega )$). These dielectric functions can be written in one formula as follows^50^:

9 $$ \varepsilon_{m} (\omega ) = \varepsilon_{m,\infty } + \varepsilon_{m,\infty } \frac{{\omega_{Lo,m}^{2} - \omega_{To,m}^{2} }}{{\omega_{To,m}^{2} - \omega^{2} - i\omega \Gamma_{m} }},\,\,\,\varepsilon (\omega ) = \left[ {\begin{array}{*{20}c} {\varepsilon_{{_{ \bot } }} } & \circ & \circ \\ \circ & {\varepsilon_{ \bot } } & \circ \\ \circ & \circ & {\varepsilon_{z} } \\ \end{array} } \right] $$

where $\omega_{To}$ and $\omega_{Lo}$ are the transverse and longitudinal frequencies of optical phonons, respectively, and $\Gamma_{m}$ is the phonon damping constant. Moreover, m = z and m = $\bot$ refer to out-of-plane and in-plane modes, respectively. The values of mentioned parameters are depicted in Table 1. Herein, the dielectric constant of SiO_2_ was set to $\varepsilon_{{SiO_{2} }} = 2.3$.

**Table 1:** Related parameters to the h-BN dielectric function.

m	$\varepsilon_{m,\infty }$	$\omega_{To,m} \,(1/cm)$	$\omega_{Lo,m} \,(1/cm)$	Γ_m_(1/cm)
z	2.95	780	830	4
$\bot$	4.78	1370	1610	5

## Numerical results

**Figure 3:**
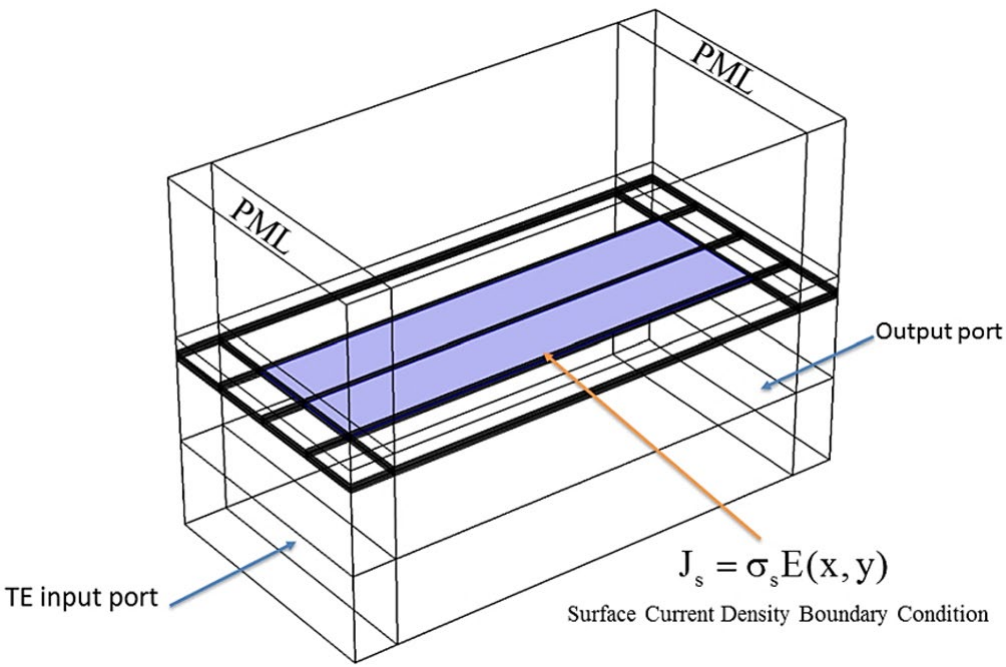
The OMOSFET configuration and boundary conditions for numerical analysis with the FEM method.

The numerical finite element method (FEM) was used to simulate the proposed device. In this case, silicene was modeled under a surface current density boundary condition of $J_{s} = \sigma_{s} \vec{E}(x,y)$, where $J_{s}$ is the surface current density and $\vec{E}(x,y)$ represents the electric field components in x and y directions. Moreover, perfect-matched layer PML boundary conditions were used for the input and output ports, according to Fig. 3.

**Figure 4:**
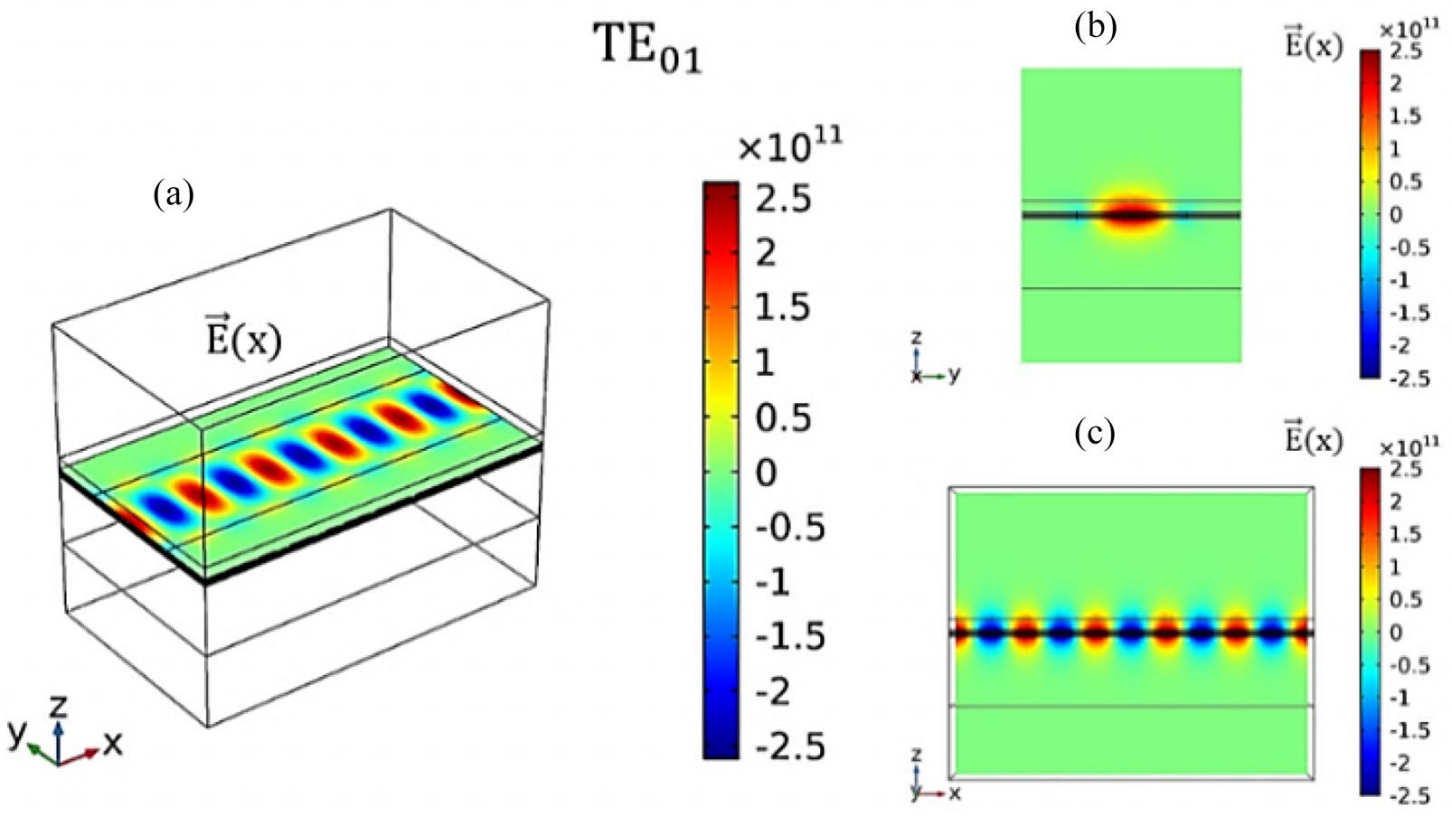
(**a**) 3D view of the electric field (${\vec{\text{E}}}$(x) (V/m)) in the TE_01_ mode at the frequency of 10 THz for W_S_ = 150 nm. (**b**) Distribution of the electric field (${\vec{\text{E}}}$(x) (V/m)) in the output port (y–z plane). (**c**) Electric field fluctuations along the length of the device (z-x plane).

**Figure 5:**
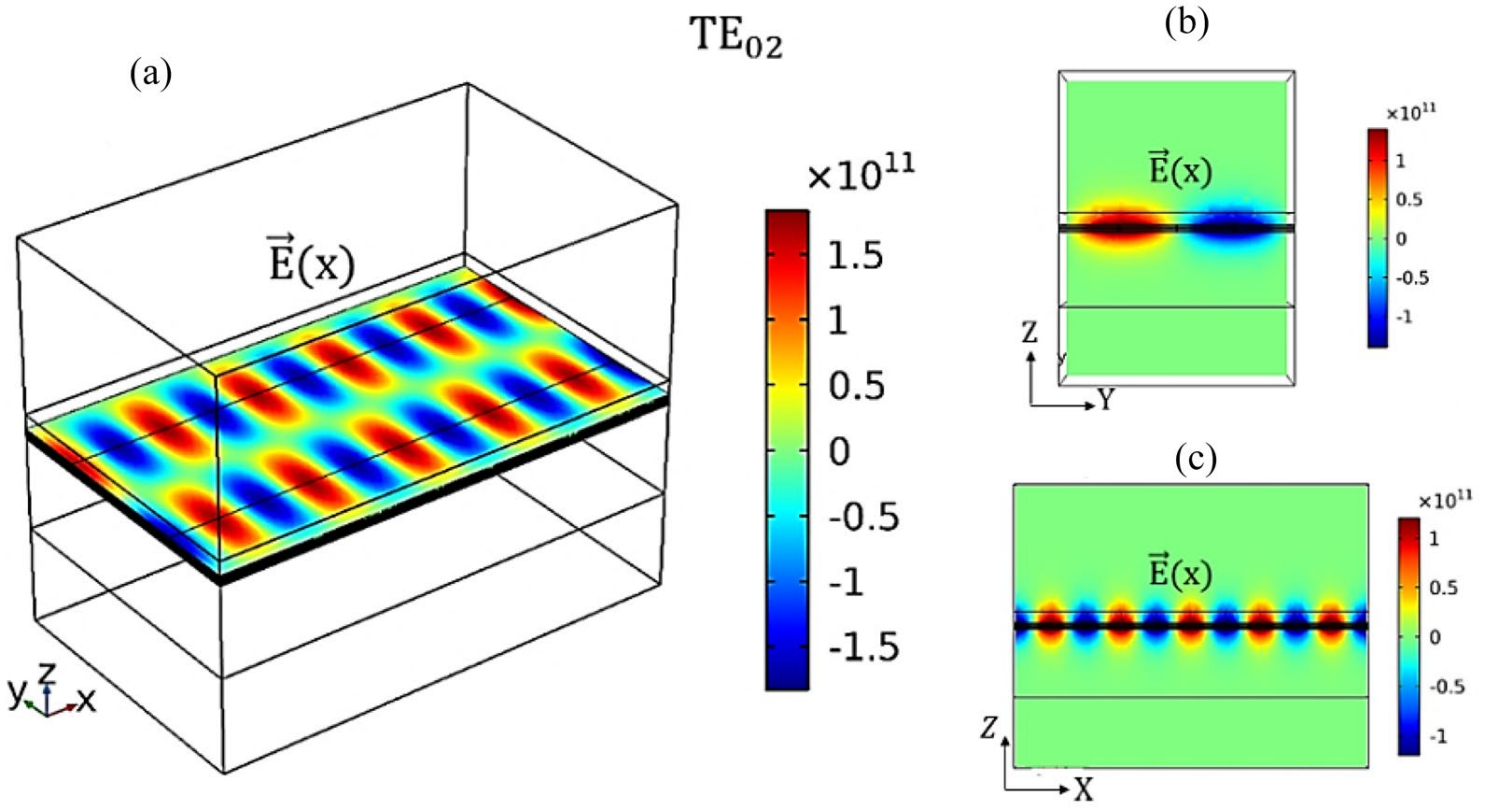
(**a**) 3D view of the electric field (${\vec{\text{E}}}$(x) (V/m)) in the TE_02_ mode at the frequency of 10 THz for W_S_ = 300 nm. (**b**) Distribution of the electric field (${\vec{\text{E}}}$(x) (V/m)) in the output port (y–z plane). (**c**) Electric field fluctuations along the length of the device (z-x plane).

Here, we investigate TE_01_ (m = 0 and n = 1) and TE_02_ (m = 0 and n = 2) emissions of the proposed device, where one-half wave and two half waves are propagated at the width of the waveguide, respectively. Figure 4 shows the profile of the electric field (${\vec{\text{E}}}$(x) (V/m)) in the TE_01_ mode at the frequency of 10 THz for W_S_ = 150 nm. In this regard, Fig. 4 (a) shows ${\vec{\text{E}}}$(x) (V/m) in 3D space obtained using E_z_ = 96.3 mV/Å at 300 K. Figure 4(b and c) shows ${\vec{\text{E}}}$(x) (V/m) at the output port and the fluctuations of the transmission wave along the longitudinal axis x–z, respectively. As well, Fig. 5 shows ${\vec{\text{E}}}$(x) (V/m) in the TE_02_ mode using E_z_ = 96.3 mV/Å and W_S_ = 300 nm at 300 K and frequency of 10 THz.

**Figure 6:**
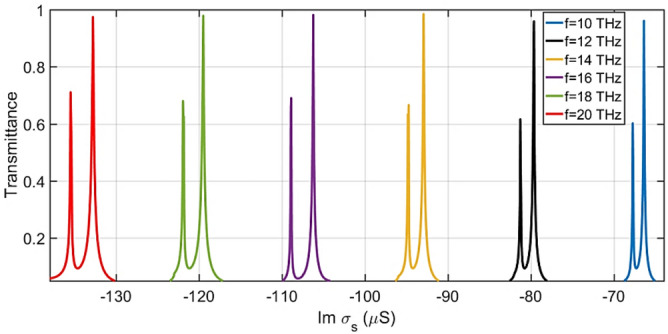
Transmission spectra calculated using Re σ_s_ = 0 and different values of Im σ_s_ for frequencies in the range of 10 to 20 THz.

The transmission spectra calculated for frequencies between 10 and 20 THz using different values of Im(σ_s_) and Re(σ_s_) = 0 is depicted in Fig. 6. As can be seen, the performance of the proposed OMOSFET remains the same for different frequencies. Each transmission spectrum has two resonances, among which the larger amplitude resonance was selected for the performance of the proposed OMOSFET. The amplitude of the selected resonance in the different frequencies is about 0.97, which is a significant value.

**Figure 7:**
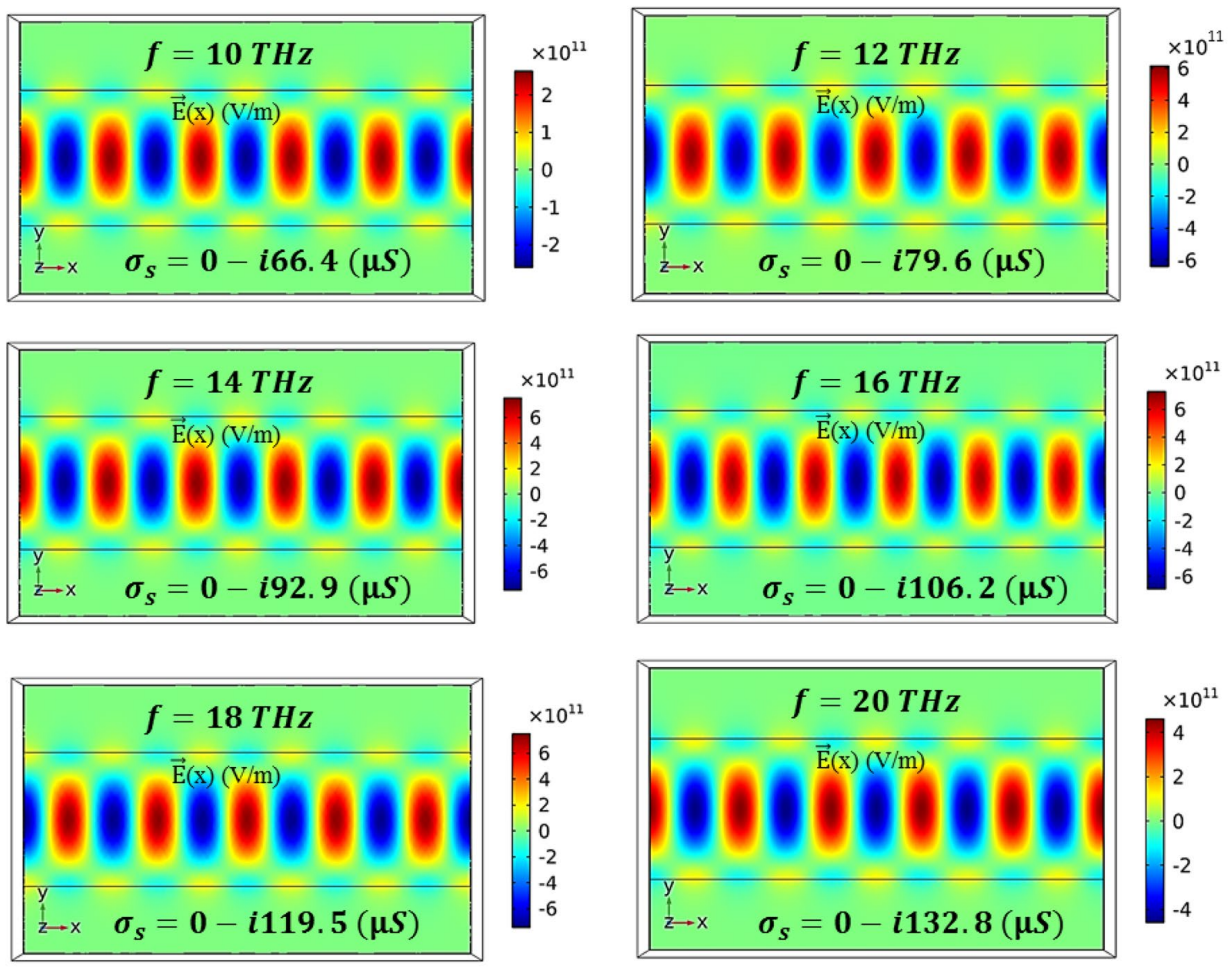
${\vec{\text{E}}}$(x) field profiles of Re σ_s_ = 0 using different values of Im σ_s_ at frequencies in the range from 10 to 20 THz.

Figure 7 shows ${\vec{\text{E}}}$(x) (V/m) at input frequencies of 10 to 20 THz. As observed, the performance of the device remains the same for different frequencies in terms of Im σ_s_ proportional to the frequency. To achieve the maximum transmission, the negative values of Im σ_s_ must also increase in proportion to the increase in the frequency. In this way, the effective value of Im σ_s_ is obtained to be 66.4 µS at the frequency of 10 THz. By doubling the frequency to 20 THz, the Im σ_s_ value is also doubled (Im σ_s_ = 132.8 µS).

Table 2 summarizes Im σ_s_, E_z,_ and V_G_ values for the maximum TE wave transmission. The values of E_z_ and Im σ_s_ were extracted from the numerical results of Figs. 2 and 6. The V_G_ values were obtained from the relationship between the electrostatic field and the voltage V_G_ = E_z_.d, in which d is the gate dielectric thickness (d = 5 nm).

**Table 2:** E_z_, Im σ_s,_ and V_G_ values for the maximum TE wave transmission.

F (THz)	10	12	14	16	18	20
Im σ_s_	− 66.4	− 79.6	− 92.9	− 106.2	− 119.5	− 132.8
E_z_ (mV/Å)	96.4	116.4	134.4	152.4	170.4	188.4
V_G_ (volt)	4.82	5.82	6.72	7.62	8.52	9.42

## Switching performance

In this section, the switching performance of the proposed device is examined. The insertion loss (IL), extinction rate (ER), and quality factor (Q) are among the most important switching parameters. IL represents the amount of power loss in the optical signal when transmitted to the output port, which is given by^51^:

10 $$ IL\left( {dB} \right) = - 10\log \left( {P_{o} /P_{I} } \right) = - 10\log T_{\max } $$

where P_o_ is the output power and P_I_ is the input power. ER is defined as the ratio of output power in the ON state to the OFF state. Ideally, no signal should be transmitted when the switch is off. The ER value is calculated from Eq. (11) ^51^

11 $$ ER\,(dB) = 10\log \left( {P_{low} /P_{high} } \right) $$

where P_low_ is the low output power in ON mode, and P_high_ is the high output power in OFF mode. On the other hand, Q is defined as follows^52^:

12 $$ Q = \frac{{F_{r} }}{FWHM} $$

**Figure 8:**
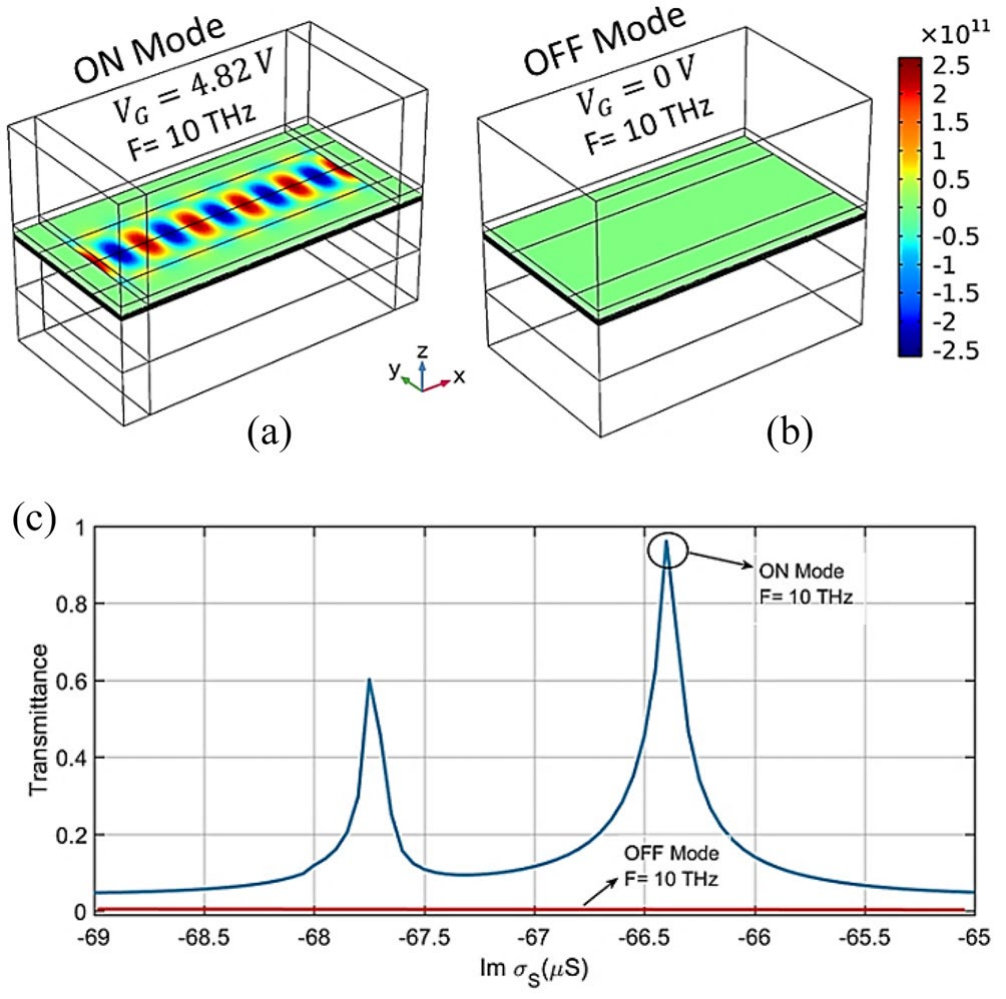
(**a**) ON and (**b**) OFF modes using V_G_ = 4.82 and 0 V, respectively. (**c**) ON and OFF mode diagrams.

Herein, F_r_ is the resonance frequency and FWHM is the full width at half maximum. Figure 8 (a) and (b) shows ON mode with V_G_ = 4.82 V and OFF mode with V_G_ = 0 V at 10 THz. The larger transmission amplitude represents the ON state, whereas V_G_ = 0 V is considered the OFF state (Fig. 8 c). As can be seen, the transmission amplitude of the input wave in the ON and OFF modes is about 0.97 and 0, respectively. According to the numerical results, the values of IL, ER, and Q for the proposed device are found to be 0.13 dB, $\infty$, and 688, respectively.

Table 3 shows a comparison between ON/OFF ratios of conventional silicene-based MOSFETs and the proposed OMOSFET. As can be seen, the proposed OMOSFET ON/OFF ratio is considerably higher due to the zero output of the OFF mode.

**Table 3:** The comparison between ON/OFF ratios of conventional MOSFETs based on silicene and the proposed OMOSFET.

References	MOSFET configuration	ON/OFF ratio
^24^	Dual-gated silicene FET	4.3
^26^	Silicene FETs with alkaline absorbers	4 × 10^8^
^34^	Z-shaped silicene nanoribbon FETs	10^6^
^35^	Silicene nanotube FET	10^7^
^36^	Li-Cl co-decorated silicene FET	4.66 × 10^4^
This work	O-MOSFET (this work)	∞

Also, in Table 4, we compare the performance of the proposed OMOSFET as an optical switch with several examples of switches based on silicene and graphene. As can be seen from the results, the proposed device has very low losses and a high Q-factor due to the zero output of the OFF mode and its sharp response.

**Table 4:** The comparison of the optical performance of the proposed OMOSFET with several graphene and silicene optical switches.

Ref	Optical switch configuration	IL (dB)	ER (dB)	Q-factor	Frequency regime
^39^	Silicene-Based Plasmonic Electro-Optical Switch	0.39	51	189	20‒50 THz
^53^	Tunable Terahertz Switches Based on Graphene Waveguide	17	∞	‒	0‒30 THz
^54^	Tunable THz Switch-Filter Based on Magneto-Plasmonic Graphene	2	51	7.8	4.8‒5.8 THz
^40^	Electro-optic switch based on graphene–silicene–graphene metamaterial	0.15	30	40	16‒70 THz
This work	O-MOSFET	0.13	∞	688	10‒20 THz

## Fabrication process

The challenges existing in the manufacture and synthesis of stable silicene have hindered the experimental development of silicene-based devices. As mentioned in the introduction section, researchers in Ref.^20^ were able to propose a method based on silicene encapsulation with Al_2_O_3_, maintaining the stability of silicene in the manufacturing and transfer processes. In addition, the authors in Ref.^55^ investigated the following three methods of silicene synthesis: i) thermal evaporation of silicene on a suitable substrate; ii) surface detachment of silicene from a substrate; and iii) intercalation through a silicide network.

**Figure 9:**
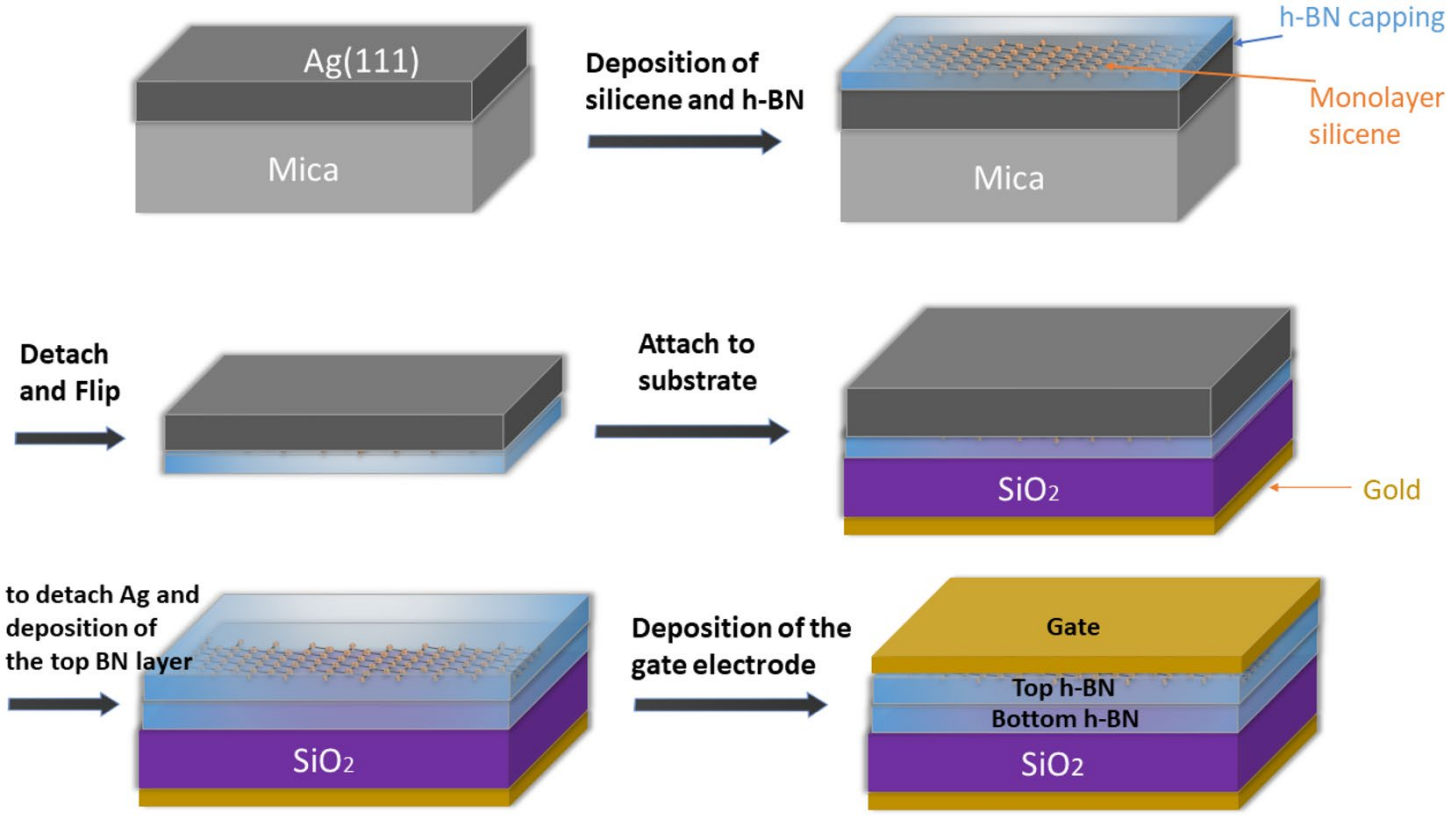
The proposed OMOSFET fabrication steps based on the method presented in Ref. ^20^.

According to the method described in Ref.^20^ and the compatibility of silicene with h-BN encapsulation^27^, we present the method shown in Fig. 9 in order to fabricate the proposed OMOSFET. Initially, an Ag (111) layer is deposited on a mica substrate, followed by depositing a monolayer of silicene and h-BN to encapsulate the silicene. In the next step, the mica layer is detached and the remaining layers are flipped, and then they are attached to the substrate of the device. Afterwards, the Ag layer is etched using a potassium-based iodine solution without damaging the underlying silicene layer^20^, and then the top h-BN layer is deposited. In the last step, the gate electrode is deposited on the device using gold.

## Conclusion

A silicene-based OMOSFET with excellent switching characteristics has been introduced and simulated using the finite element method. The TE-polarized input light of this OMOSFET was controlled by the gate terminal embedded at the top of the structure. The switching characteristics of the proposed device are nanometer dimensions, ultra-low insertion losses of 0.13 dB, infinite extinction ratio, and a quality factor of 688. Due to these excellent characteristics, this device can be an excellent candidate for optical integration soon.

## Data Availability

The datasets used and/or analyzed during the current study are available from the corresponding author on reasonable request.
